# New Directions and Challenges in Targeted Therapies of Advanced Bladder Cancer: The Role of FGFR Inhibitors

**DOI:** 10.3390/cancers14061416

**Published:** 2022-03-10

**Authors:** Katarzyna Szklener, Paulina Chmiel, Adam Michalski, Sławomir Mańdziuk

**Affiliations:** Department of Clinical Oncology and Chemotherapy, Medical University of Lublin, 8 Jaczewskiego Street, 20-090 Lublin, Poland; gp.chmiel@gmail.com (P.C.); michalski.g.adam@gmail.com (A.M.); slawomir.mandziuk@umlub.pl (S.M.)

**Keywords:** bladder cancer, fibroblast growth factor receptor, immunotherapy

## Abstract

**Simple Summary:**

The aim of this study was to present and analyze the up-to-date literature describing the epidemiology, genetics, and histopathology of bladder cancer, as well as the latest methods of bladder cancer treatment. The treatment of urothelial cancer was divided depending on its stage and advancement. FGFR3 mutations and overexpression occur more frequently in bladder cancer than any other malignancy, occurring in nearly 80% of the tumors. Closer acknowledgement of targeted therapy will help physicians to navigate specific groups of patients for whom this treatment strategy can be beneficial. To that end, intense clinical research was conducted, bringing evidence for effectiveness and safety of FGFR inhibitors. Recent years of research have truly set a positive perspective for the better understanding of the complex issue of urothelial carcinoma pathology and management.

**Abstract:**

Bladder neoplasms, including the most common urothelial carcinoma, have been an escalating problem for years, especially in highly developed countries. Recent decades have brought us a steadily growing share of this cancer in terms of both morbidity and mortality statistics. Bladder neoplasms are not only a therapeutic challenge but also an economical one due to the demanding, costly diagnostics and treatment. The treatment of urothelial cancer can be divided depending on the stage and advancement; thus, we can distinguish three main categories: non-muscle invasive bladder cancer, conventionally treated by surgical interventions; muscle invasive bladder cancer, conventionally treated with chemotherapeutics; and advanced bladder cancer with distant metastases, conventionally treated with the intensive chemotherapy in the MVAC scheme (methotrexate, vinblastine, doxorubicin, and cisplatin). Recent years have brought a breakthrough: immunotherapy and targeted therapy were discovered to be beneficial for patients disqualified from chemotherapy or patients who progressed despite treatment. This literature review summarizes the latest research into the use of targeted therapy in the treatment of advanced bladder cancer, its benefits, and its limitations.

## 1. Introduction

In 2020, GLOBOCAN 2020 reported an estimated 19.3 million new cancer cases diagnosed worldwide. Among the statistics, bladder cancer (BC) ranks as 10th most often detected malignancy and is responsible for over half a million cases and two hundred thousand deaths [1]. In Poland, it is prevalent in 6.7% of cases in men and 2% of cases in women, while also standing as the fifth cause of cancer deaths among men [2]. Worldwide statistics differ significantly, with BC accounting for 4.5% of cancer cases in men, being classified as “other cancer” in females and being the ninth cause of cancer deaths in males [1]. However, there has been a downward trend in the incidence of BC over the last 40 years [3]. The most common BC is urothelial carcinoma, a cancer derived from the transitional epithelium of the urinary tract, which appears in 90% of cases. The remaining 10% of cases consist of squamous cell carcinoma, accounting for 5%, and other tumors, including sarcomas and metastases of other neoplasms, accounting for 5% [4]. BC is found mainly in patients over 50 years of age, and the mean age of the onset in the United States is 73 years [5]. Urothelial carcinoma is most often found in highly developed societies, mainly in Western and Southern Europe as well as North America. However, in African countries, squamous cell carcinoma of bladder, associated mainly with the carcinogenic effect of schistosomes, is prevalent [6,7,8,9]. BC incidence has been linked to various risk factors as well as a country’s level of development expressed in GDP [4]. Several risk factors of BC have been discovered: tobacco smoking, exposure to arsenic, and exposure to aromatic amines (2-naphthylamine, 4-aminobiphenyl and benzidine) and 4,4′-methylenebis(2-chloroaniline) [10,11,12]. Out of those three, smoking is arguably the most important one due to the prevalence of tobacco use worldwide. Furthermore, smoking is also responsible for the occurrence of bladder tumors at an earlier age [13]. Looking at the incidence of urothelial cancer in developed countries, an obvious conclusion was drawn about its correlation with the toxins present in the environment, especially the aforementioned aromatic amines and 4,4′-methylenebis(2-chloroaniline) found in both tobacco smoke and several items such as hair dyes, paints, and fungicides, among others [4,12]. To sum up, BC is a significant medical and economic problem in highly developed countries. What is more, an appropriate treatment based on modern immunotherapy and targeted therapy has allowed a significant extension of patients’ lives and reduction in mortality in recent years [1].

## 2. Current Treatment Guidelines in Advanced Bladder Cancer

Although the treatment of urothelial cancer largely depends on its clinical stage, the decision between surgical treatment and pharmacological therapy depends on the type of neoplasm. Advanced neoplasms infiltrating the bladder muscle usually require radical cystectomy with a resection of regional lymph nodes [14]. Bladder-sparing treatment is possible, but it is a significantly more challenging approach requiring much greater involvement of both a multidisciplinary team of specialists and the patient [15]. Despite the properly performed resection, the risk of recurrence and metastasis in patients with MIBC is close to 50% due to the presence of micrometastases in the surrounding tissues [16], which indicates the need to consider additional therapies in advanced forms of this cancer. The benefits of using cisplatin in this indication were demonstrated as early as the 1970s [17], while studies from the 1980s brought a breakthrough regarding a significant downstaging of MIBC and a reduction in the risk of recurrence associated with neoadjuvant chemotherapy [18]. In total, 5% of patients diagnosed with metastatic BC receive chemotherapeutic-based treatment as a standard [19]. Advanced urothelial carcinoma is considered an incurable disease, and the only currently available option for the affected patients is palliative care [16]. Current state of knowledge supports the use of chemotherapy in advanced/metastatic disease, providing a reliable symptom relief and a median survival ranging from 13 to 18 months [14]. Currently, according to the guidelines of the European Urological Association, ECOG-assessed patients with metastatic disease should be treated with cisplatin-based chemotherapy. Patients ineligible for cisplatin should receive immunotherapy (PD-L1 positive patients) or carboplatin (PD-L1 negative patients). Javelin Bladder 100 showed significant advantage of avelumab as maintenance therapy post chemotherapy. The primary endpoint was overall survival, which was prolonged to 21.4 months conversely to the control group in which overall survival was 14.3 months [20]. Pembrolizumab is recommended as the second-line treatment of metastatic disease [21]. Treatment with platinum derivatives has for many years remained the standard of care for patients with advanced urinary carcinoma. Nevertheless, numerous cases not qualifying for this therapy prompted further search for the optimal method. We are currently experiencing the heyday of immunotherapy in the treatment of the urinary bladder, as evidenced by numerous successful clinical trials, including CheckMate275 and Javelin Bladder 100. Immunotherapy is directed at the immune checkpoints, currently based on PD-L1 inhibitors [22]. Recent years and pathogenetic analyses of urothelial cancer have also allowed the use of targeted therapy based on FGFR3 inhibitors.

## 3. An Overview of the Bladder Cancer

BCs rarely have a family background; most of them occur as a result of chronic irritation and inflammation resulting from exposure to harmful environmental factors [23]. Invasive BCs develop on the basis of precancerous lesions—CIS (carcinoma in situ) or non-infiltrating papillary urothelial carcinoma. Several concepts for carcinogenesis of BC have been described, but each of them includes deletions within chromosome 9 and mutations within the TP53 gene [24]. The basic classification of BC, which determines the therapeutic approach, includes non-infiltrating cancers—nMIBC (non-muscle invasive bladder cancer) and infiltrating cancers—MIBC (muscle invasive bladder cancer), with 70% of diagnosed BCs belonging to the nMIBC group [25]. Histological classification of urothelial carcinoma is complex, but a proper assessment is crucial, as variants of urothelial carcinoma differ significantly in their level of aggressiveness. Indeed, we can distinguish the luminal papillary, luminal, luminal infiltrated, basal/squamous, and neuroendocrine-like variants, all of which demand specific treatment. Essentially, a tumor in one patient might consist of numerous types of histological weaving [23]. As a result of the increasing importance of targeted therapies and immunotherapy, research on the genome of urothelial neoplasm has been recently started. Therefore, the identification of dominant and clinically significant mutations of the neoplasm has become possible. The Cancer Genome Atlas study identified 32 statistically significant gene mutations in urothelial carcinoma, with the majority of them occurring in genes controlling the cell cycle and repair within the cell’s genome [24]. As discovered, MIBCs are mainly characterized by mutations within the TP53 and RB1 genes [26]. One of the main mutations that influences the clinical approach is a mutation in the FGFR3 gene. Histological types of bladder cancer have different mutation profile, which implicates the best therapy depending on the type. The luminal-papillary subtype presents significant changes in FGFR3, luminal-infiltrated has high expression of myofibroblast genes and immunological markers. The highest expression of immunological markers has been found in the basal/squamous subtype [27]. On this note, the knowledge of specific histological subtype in combination with clinical stage allows us to choose the best and most suitable treatment for the patient [28].

## 4. FGFR in Bladder Cancer

**Figure 1:**
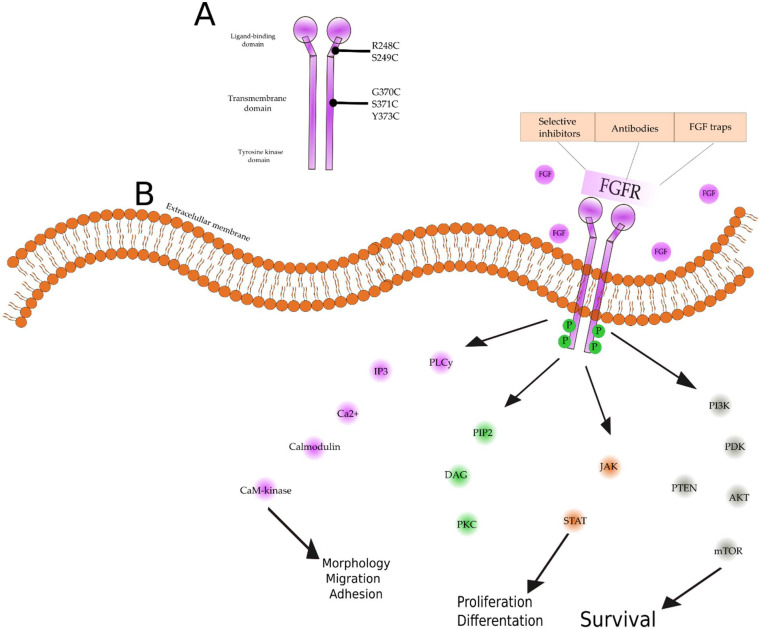
(**A**) Structure of fibroblast growth factor receptor and main mutations found in advanced bladder cancer. FGFRs are tyrosine kinase receptors, consisting of a heparin-binding sequence and immunoglobulin-like extracellular sequence and a hydrophobic transmembrane domain and intracellular tyrosine kinase domain [37]. Most common genetic changes are missense mutations and FGFR3-TACC3 fusion, primarily in ligand-binding domains (R248C and S249C), less frequently in the transmembrane domain (G370C, S371C and Y373C), rarely in the tyrosine kinase domain [27,37]. Alterations lead to overexpression and hyperactivation of FGFR. (**B**) The FGF signaling pathway. Ligand binds to an FGFR monomer, which leads to dimerization and intracellular phosphorylation, resulting in conformational changes. This provides the means to start signaling pathways for FGFRs. Activated FGFRs phosphorylate FRS2, which opens the way for PI3K, AKT, mTOR, or the RAS/RAF/MEK/MAPK cascade. Activated FGFRs also phosphorylate JAK kinases, which lead to STAT activation. FGFRs can also recruit and phosphorylate PLCγ, thereby initiating signaling through the DAG/PKC or IP3-Ca^2+^. All of those pathways have a crucial role in tumor development [25,27,38,39,40]. FGFRs (fibroblast growth factor receptors), FRS2 (fibroblast growth factor receptor substrate 2), PI3K (phosphoinositide 3-kinase), AKT (protein kinase B), mTOR (mammalian target of rapamycin), MAPK (mitogen-activated protein kinase), JAK (Janus kinase), STAT (signal transducer and activator of transcription), PLCγ (phospholipase C gamma), DAG (dystroglycan), PKC (protein kinase C), IP3 (inositol trisphosphate).

Family of fibroblast growth factor receptors (FGFRs) are tyrosine kinase receptors playing an important role not only in oncogenesis but also in the wild range of cell signaling pathways. From the early stages of embryonic development, they are responsible for key cellular interactions and functions [29,30]. In classic overview, there are four FGFR (FGFR1–FGFR4) tyrosine kinases and eighteen FGF ligands that activate them. FGFRs are formed with three extracellular binding ligand domains, a transmembrane domain, and an intracellular domain, which is tyrosine kinase [31]. Depending on the tissue extracellular domain D3, it can alternatively be spliced and formatted as epithelial (“b” form) or mesenchymal (“c” form) [31,32]. Additionally, recent studies indicate the occurrence of FGFR5/FGFRL1 with unknown function and different morphology [33,34]. Activation of the receptor is determined by tissue downstream signaling through the RAS–MAPK and PI3K–AKT pathways’ calcium ions and PKC [35]. Final effects of the pathways are individual for the cell and include mitogenesis in the MAPK pathway, cell survival in the PI3K pathway, and mobility in PKC [36] (Figure 1).

## 5. Fibroblast Growth Factor and Its Receptor in Bladder Cancer

At the base of every carcinogenesis lies various genetic changes depending on the tumor. Numerous mutations and genome instability enable the formation of mass and the microenvironment of the tumor. They are distinguished by functional changes [41]. These changes include escaping immune surveillance, avoiding growth inhibitors, and resisting cell death signals [41]. According to the current state of knowledge, there is a need to focus on those features whose blocking will enable the effective treatment of the cancer. Thus, we developed immunotherapy based initially on BCG and now more and more often on PD-1 and PD-L1 blockade [41,42]. Intensive research on disorders of the genome of urothelial carcinoma proves the correctness of directing research towards FGFR inhibitors therapy [43]. Fibroblast growth factor (FGF) belongs to the family of growth factors which, by binding to fibroblast growth factor receptors 1–4 (FGFR1–4) tyrosine kinases, activates numerous intracellular processes and pathways. It has been proven that the FGFR enhancement mutation occurs in various cancers, including breast, bladder, and leukemia [44]. FGF and FGFR are involved in processes such as proliferation, migration, and cell shaping [44]. Various genetic disorders affect the oncogenic function of FGFR, as evidenced by specific gene amplification, activating mutations, and chromosomal translocations [25,45]. BC is characterized by one of the most frequent mutations in the FGFR1–4 genes [24,44]. Point activating mutations in the FGFR3 gene are found in approximately 80% of urothelial carcinomas [46] and in 15% of MIBC cancers [47]. However, a mutation in the FGFR3 gene appears much more frequently in low-grade tumors [48]. Urothelial carcinomas and their metastases, in which this mutation was discovered, respond well to targeted therapy with FGFR3 inhibitors [23]. Mutations in the FGFR3 gene might also be associated with a better prognosis, especially in nMIBC, where they indicate a lower probability of progression to MIBC [23]. Currently, comparative studies of various neoplasms clearly indicate a high percentage of FGFR mutations in BC, although they are not found in other tobacco-dependent neoplasms, such as lung cancer or esophageal cancer [49]. Moreover, in urothelial carcinoma, mutations in the chromatin regulatory genes have been detected significantly more frequently than in any other neoplasm, which indicates a possibility of new therapeutic directions [24]. Mutation studies of BCs discovered a correlation between the type of mutation and the stage of the disease: FGFR3 mutations were discovered to be much more common in low-stage and low-grade cancers, while in advanced cancers associated with exposure to tobacco smoke, TP53 mutations have been significantly more frequent [50]. Both wild-type and mutant FGFR3 are expressed on tumor cells, suggesting that overexpression plays the main role in the tumorigenesis, not particularly the mutation [51]. On the other hand, there is strong evidence that overexpression of wild-type FGFR3 was correlated with high-stage tumors [52]. The same study questioned mutation and expression association with tobacco, sex, and age. As a result, no significant correlation was found [51]. In some studies, there were several cases of different mutations of FGFR3 depending on the precise localization of the tissue. Correspondingly, deeper parts of the tumor seem to have a more aggressive wild-type of the mutation [53]. There is little evidence about the role of FGFR3 in advanced or metastatic bladder cancer. Most of the data do not see the correlation as being as strong as in the NMIBC, but expression of the protein is found in metastatic BC, and some studies suggest finding it both in the primary tumor and metastases [54]. The exact pathway of bladder cancer carcinogenesis in correlation with FGFR3 is not established, yet some data showed that FGFR3 mutations suppressed the inflammatory response in early stages and that further inflammation was corelated rather with progression than with FGFR3 mutation [55]. At the same time, FGFR3 can also be used in the diagnosis of recurrent BC in patients with low-stage tumors [26]. As it appears, FGFR3 mutations are not only limited to targeted therapy in urothelial carcinoma. Lately, it was indicated that FGFR3 pathways can be correlated with a poor response to immunotherapy in bladder cancer. This mutation is the most common in the non-inflamed type of tumors. Accordingly, the activation of the receptor leads in some ways to deactivation of T cells in the microenvironment of the tumor [56]. In addition to resistance to immunotherapy, the implicating role of FGFR3 phosphorylation of EGFR in cisplatin resistance is observed [57]. Then again, the study PURE-01 did not find strong enough evidence of the FGFR3 mutation in resistance to a PD-1 inhibitor in patients with muscle invasive bladder cancer [58]. In light of all the studies, we can distinguish the importance of FGFR3 genetic alterations in bladder cancer. The relative specificity, multidirectional research, and the role of FGFR in BC initiated studies focused on the treatment of BCs with FGFR inhibitors. As of now, the only drug in this group currently approved by the FDA is erdafitinib used in patients with advanced BC. Apart from erdafitinib, other FGFR inhibitors such as pemigatinib, rogaratinib, infigratinib, derazantinib, futibatinib, dovitinib, nintedanib, vofatamab, anlotinib, debio 1347, BIBF-1120, and AZD4547 are being actively researched and developed [16] (Table 1).

## 6. Drug-Evaluating Studies

### 6.1. Erdafitinib

Erdafitinib (JNJ-42756493), an FGFR1–3-inhibitor, is the first drug of this group approved as a treatment option in urothelial cancer. It is registered for use in patients with locally advanced or metastatic urothelial cancer susceptible to genetic changes in FGFR3 or FGFR2 who progressed during or after platinum chemotherapy, including within 12 months after platinum-containing neoadjuvant or adjuvant chemotherapy [59]. The oral inhibitor showed a strong and dose-dependent antitumor effect in cell cultures [60]. Currently, many clinical trials are being conducted involving erdafitinib. One of them, described by Loriot et al., is a phase II trial evaluating the efficacy and safety of this agent in patients diagnosed with inoperable or metastatic urothelial carcinoma [59]. The study required them to have disease that was measurable according to the criteria for evaluating solid tumor response (RECITS 1.1) at the beginning of the process and adequate kidney, liver, and bone marrow function, as well as performance status as assessed by ECOG (Eastern Cooperative Oncology Group) less than or equal to 2. Disease progression, as assessed by RECIST 1.1, subsequent to prior chemotherapy, was also a prerequisite [59]. The study also included so-called “chemoresistant participants” who were selected by researchers based on their progression in RECIST 1.1 criteria 12 months after the last treatment dose. Patients with disease progression according to RECIST 1.1 and treated with anti-PD-1/PD-L1 antibodies were also qualified. The primary endpoint of the study was the proportion of participants with the best overall response (time frame—1 year), defined as the proportion of participants with measurable changes who achieved a complete response (CR) or partial response (PR) based on the response assessment criteria for solid tumors in version 1.1 (RECIST v1.1). Secondary endpoints, apart from treatment effectiveness, also include its safety. The secondary endpoints were progression-free survival (assessed up to 5 years); duration of response, as defined by the earliest date the participant obtained CR or PR; overall survival (OS); and number of participants with adverse events (AE) and serious adverse events (SAE). The percentage of biomarker-positive patients (the presence of circulating biomarkers—DNA, RNA or proteins associated with FGFR aberrations) was also assessed. The safety profile was assessed based on secondary endpoints—plasma erdafitinib concentration, plasma erdafitinib clearance, and volume of distribution of erdafitinib. At the same time, a study focused on the interaction of erdafitinib with other DDI preparations was carried out, investigating drugs strongly influencing enzyme metabolism: midazolam, which is a sensitive cytochrome P450 substrate, and metformin, as a substance strongly activating cationic transporters. As secondary endpoints of the study, the interaction was tested with the concentration of midazolam and its metabolites as well as with metformin. Preliminary results of the clinical trial revealed that the use of erdafitinib in patients with FGFR changes was associated with an objective response (OR) in 40% of patients. The median progression-free survival (mPFS) was 5.5 months, and the median overall survival (mOS) was 13.8 months. Response rates were similar regardless of the patients’ previous treatment regimens but also regardless of characteristics such as age, gender, and assessment of renal function. In total, 46% of patients reported treatment-related serious adverse events; the most common adverse events (AEs) in this group were hyponatremia, asthenia, gastritis, hyperphosphatemia, and dry mouth. A total of 21% developed retinopathy associated with FGFR inhibitors. In 77% of patients, hyperphosphatemia was reported, which results from the mechanism of FGFR inhibitors and is the most common AE, yet frequency decreased during treatment. Hyperphosphatemia is associated with FGFR1, a key factor in phosphate homeostasis. The main cause of this is the inhibition of renal excretion of phosphate—and moreover, an inhibition of FGFR23—which leads to increased levels of vitamin D, which retrenches phosphate [61]. Most of the side effects from treatment were transient and resolved after dose reduction or adjustment of erdafitinib. Based on the results, we can conclude that erdafitinib has significant antitumor effects in patients with advanced urothelial carcinoma and specific changes in the gene for FGFR. Taking into account the emerging adverse events and treatment benefits, we can confidently say that regardless of the previous treatment, therapy with erdafitinib has a positive effect on the treatment of patients. Based on this clinical study (NCT02365597), the FDA approved erdafitinib for the treatment of adult patients with metastatic urothelial carcinoma and significant gene changes in FGFR3 or FGFR2.

Alifrangis et al. described a phase III study focused on the efficacy of erdafitinib compared with vinflunine, docetaxel, or pembrolizumab in patients with advanced BC with selected FGFR gene aberrations who have progressed despite one or two stages of treatment (including one with steps that had to include anti-PD-L1 immunotherapy) [62]. Patients with histologically confirmed urothelial carcinoma requiring a change in treatment due to the disease progression and with appropriate genetic profiles and organ function (kidneys, liver, bone marrow) were eligible for the study [62]. The primary endpoint of the study was overall survival (OS) within approximately 3 years [62].

### 6.2. Pemigatinib

Described in a 2017 phase II study, FIGHT-201 sought to evaluate the efficacy and safety of pemigatinib in patients with advanced urothelial carcinoma [63]. Researchers aimed to assess the overall response rate (ORR) of pemigatinib monotherapy in patients with advanced or inoperable cancer and appropriate FGFR3 aberrations [63]. Three patient cohorts were established to test the optimal mode of administration (intermittent dose/continuous dose) [63]. Adult patients with histologically documented urothelial carcinoma, changes in the FGFR3 gene, and radiologically measurable tumors according to RECIST 1 were enrolled in the study [63]. The primary endpoint of the study was ORR in patients with FGFR3 mutations based on RECIST 1.1 [63]. Secondary endpoints included the safety and tolerability of pemigatinib based on the frequency, duration, and severity of adverse events; ORR measuring the efficacy of pemigatinib in patients with advanced/metastatic or unresectable urothelial carcinoma with different molecular subsets; mPFS from RECIST 1.1; and duration of response (days from first documented response to disease progression or death) [63]. ORR was 25%, and the observed adverse events were diarrhea, fatigue, and alopecia in both studied cohorts, while hyperphosphatemia was observed in more than half of the patients [63]. The FIGHT-205 study is also currently being conducted, taking into account pemigatinib monotherapy compared with combination therapy with pemigatinib and pembrolizumab in a given indication [64].

### 6.3. Rogaratinib

Rogaratinib is a strong and selective FGFR inhibitor, which has already been proven to exhibit significant antitumor activity in several in vitro studies focused on BC, lung cancer, or head and neck cancers [65]. Currently, to confirm these observations, many clinical trials are being conducted, including the FORT-1 study comparing rogaratinib with chemotherapy in FGFR positive patients with advanced urothelial carcinoma [66]. Patients with histologically proven urothelial carcinoma whose tumors were FGFR-1 or FGFR-3 positive and who had received at least one course of platinum-based chemotherapy in the past and whose disease had progressed were enrolled in the study [66]. The primary endpoints of the study were ORR; secondary endpoints were mPFS and the incidence of adverse events reflecting safety [66]. The evaluation time frame was up to 45 months. ORR was 19.5% and 19.3% (*p* = 0.56), mPFS was 2.7 (95% CI) and 2.9 (95% CI) months with rogaratinib and chemotherapy, respectively [66]. Grade 3–4 AEs were observed in 47% of subjects with rogaratinib and 56% with chemotherapy [66]. In addition, among FGFR-3 positive patients, ORR values for rogaritinib and chemotherapy were 52.4% and 26.7%, respectively [66]. FORT-2 additionally extends the scope of research on rogaratinib, checking the use of the preparation together with atezolizumab in patients disqualified from receiving platinum chemotherapy. The initial research results are extremely promising [67].

### 6.4. Infigratinib

Oral infigratinib is undergoing a clinical trial as adjuvant therapy in patients with invasive urothelial carcinoma harboring susceptible FGFR3 alterations—PROOF 302 [68]. Patients in this study show a high risk of disease recurrence with operation alone, are ineligible for cisplatin adjuvant therapy, or show residual disease after chemotherapy [68]. Infigratinib at the dose of 125 mg was compared with placebo. The primary endpoints of the study were disease-free survival (DFS) centrally reviewed, analyzed via stratified log-rank test, Kaplan–Meier method (Brookmeyer–Crowley CI), and Cox model (hazard ratio) [68]. The primary endpoints include safety and tolerability of therapy and OS [68].

### 6.5. Futibatinib

Futibatinib (TAS-120) is a highly selective FGFR1–4 inhibitor under ongoing trial in patients with various malignant tumors. Lately, some positive results in cholangiocarcinoma treated with TAS-120 have been found. A first-in-human trial, FOENIX-1, enrolled 86 patients with FGFR aberrations to evaluate the safety of the substance. The trial enabled the definition of a 20 mg daily dose, and the most common treatment-emergent adverse (TEAEs) events were hypophosphatemia, diarrhea, and constipation; as for antitumor activity, 41 patients experienced stable disease and 5 of them a partial response [69]. The FOENIX-CCA2 phase II trial was focused on patients with advanced solid tumors, including urothelial carcinoma, who progressed after first-line therapy and harbored the FGFR2 mutation. The primary endpoint was objective response rate (ORR) based on independent radiology review. The drug safety profile was also examined. The ORR was 34.3% and even one patient achieved complete response (CR) after a follow-up PFS of 7.2 months was achieved. The most common adverse event was hyperphosphatemia manageable with diet and medications [70]. Other studies have started recently and, according to the available data, are still ongoing. The FOENIX-CCA3 phase III trial is focused on patients with cholangiocarcinoma and FGFR2 gene mutations. The study aims to compare an FGFR inhibitor with chemotherapy based on gemcitabine and cisplatin. The primary endpoint is PFS, while secondary endpoints are ORR, OS, and safety (NCT04093362). Another current study concerns a combined futibatinib therapy with pembrolizumab in advanced or metastatic urothelial carcinoma (NCT04601857). The aim is to evaluate safety and antitumor activity in this combination, especially in light of PD-1 resistance in specific tumor samples. The primary outcome is objective response rate, and the secondary outcome measures are PFS, OS, and adverse events. Both FGFR mutant and wild-type patients are included.

### 6.6. Dovitinib

Dovitinib is an FGFR1-3 inhibitor with less effect on FGFR4. By occupying the ATP-binding region, dovitinib stops the downregulation of FGFR pathways. Recently, the phase II trial of dovitinib in patients with progressive advanced urothelial carcinoma was ongoing. Patients included had FGFR-3 mutated or wild-type cancer, and all of them progressed on platinum-based or combined therapy. The trial was terminated because of the ORR not meeting criteria to continue. Despite being well tolerated and potentially advisable in tumors with this mutation, dovitinib did not show sufficient activity [71].

### 6.7. Derazantinib

We are currently experiencing the heyday of modern anticancer therapies. The methods developed in recent years have brought a breakthrough after almost thirty years of classic chemotherapy, radiotherapy, and surgical treatment. In the last decade, the pathogenetic mechanisms of UC have also been thoroughly investigated [24,71]. The new approaches assume penetrating inside the cell and stopping the neoplastic process at its base at the stage of mutating the genome of a single cell. The first and main method of treating urothelial cancer to this day remains therapy based on platinum derivatives [25]. It is impossible to forget about the intensively developing immunotherapy of bladder cancer, which currently offers hope for patients resistant to chemotherapy and patients with absolute contraindications to the use of platinum-based agents [72]. Currently, targeted therapies are within the therapeutic range, including FDA-approved erdafitinib used in the treatment of advanced bladder cancer after platinum treatment progression [60]. Intensive clinical trials also prove the effectiveness of other preparations in this indication. Comparing targeted therapy with chemotherapy has often shown its safe nature and few adverse events associated with the treatment. The most severe complication of this therapy seems to be retinopathy, which must be addressed in further clinical trials in order to determine safe doses of targeted therapy. The effectiveness of FGFR inhibitors has been proven in various neoplasms in which a mutation of a given gene has been found. However, it is imperative to extend the research to tumors without mutations and to evaluate the efficacy of targeted therapy in combination with immunotherapy. The first clinical trials show only the effectiveness of interfering with the tumor by these preparations; it seems necessary to precisely determine the optimal method, dose, and duration. We believe that currently available data serve as a rationale for a therapy composed of novel, targeted therapies in combination with currently established medication. Undoubtedly, this is an impressive step forward in understanding the mechanism of tumor growth and stopping it locally, without interfering with the overall well-being of the patient.

### 6.8. Vofatamab

Vofatamab (B-701) is a novel monoclonal antibody specific for FGFR-3 used in trials with stage IV locally advanced or metastatic urothelial carcinoma. Patients with histologically proven urothelial carcinoma whose tumors were FGFR-3 positive and who had received at least one course of platinum-based chemotherapy in the past and whose disease had progressed were enrolled in the phase Ib/II study showing efficiency of vofatamab with or without docetaxel (FIERCE-21) [73]. The primary endpoint of the study was PFS, and the evaluation time frame was 3–4 years. Safety and dose were determined. Phase Ib showed that vofatamab was well-tolerated with expected myelosuppression [74]. Major activity was seen in patients with mutant FGFR-3 compared with wild-type patients. Significant AEs were asthenia, diarrhea, and flushing. Unlike other FGFR inhibitors, no hyperphosphatemia was reported since vofatamab has a different mechanism of action [75]. In the phase Ib/II FIERCE-22 study, patients received vofatamab with pembrolizumab, and ORR was 36% overall; furthermore, biopsies were conducted and showed that treatment induced immunological changes with inflammatory response [76]. As for now, data are still limited, but the above mentioned trials showed promising results. The key finding seems to be the elimination of the main AE, which in other inhibitors was hyperphosphatemia.

## 7. FGFR Resistance and Further Directions

The major drawback in achieving therapeutic success is targeted drug resistance. In most cases, it is acquired resistance, which means resistance that occurred during treatment. It can be associated with the selection of existing favorable alterations, acquisition of new mutations, modification of the target, or engaging another survival factor [76,77,78,79,80]. Beyond that, the tumor microenvironment can affect tumor cell survival during treatment [81]. In FGFR-mutated leukemia, activating the V561M mutation in the FGFR1 kinase domain and inactivation of PTEN resulted in resistance to both FGFR inhibitors and nonspecific inhibitors such as AZD4547, BGJ398, ponatinib, TKI258, and E3810 [82]. Simultaneously, mutation of gatekeeper residue (V555M) of FGFR3 can be the reason for resistance, as shown in human tumor cell lines [83]. FGFR signaling is a part of many pathways and is shared by many tyrosine kinases, including EGFR and VEGFR, which allows a tumor to escape from targeted treatment via enhancement of the remaining mechanisms [84]. Screening of urothelial carcinoma cell line RT112 (FGFR3-TACC3 translocation) displayed PI3K, PI3K-protein kinase B, or EGFR pathway as the cause of resistance [85]. The highly sensitive for FGFR inhibitors lung cancer cell lines DMS114 and H1581 consistently showed vitality despite the targeted treatment, which suggested the existence of a resistant sub-population. After the research was completed, the sustained MAPK pathway activation was specified to be the cause of resistance [86].

A crucial need for the future is finding a solution for overcoming the resistance. In vitro assays indicated that sulfated polysaccharide of *Sepiella maindroni* ink overcomes resistance by decreasing AKT phosphorylation and downregulating CDK4, MMP2, and Bcl-2, causing a reduction in cells’ viability and migration [87]. Both in vitro and in vivo studies showed that the new third-generation FGFR1 inhibitor GZD824 overcomes FGFR1-V561F/M mutant resistance, which is strong evidence of the benefit for patients who did not respond to older generations of inhibitors [88]. In light of that information, we believe that more research is needed.

## 8. Conclusions

We are currently experiencing the heyday of modern anticancer therapies. The methods developed in recent years have brought a breakthrough after almost thirty years of classic chemotherapy, radiotherapy, and surgical treatment. In the last decade, the pathogenetic mechanisms of UC have also been thoroughly investigated [24,71]. The new approaches assume penetrating inside the cell and stopping the neoplastic process at its base at the stage of mutating the genome of a single cell. The first and main method of treating urothelial cancer to this day remains therapy based on platinum derivatives [25]. However, this kind of systemic treatment has prospective alternatives, fit both for the purpose of first- and second-line treatment, including intensively developing immunotherapy. In the case of bladder cancer, immunotherapy currently offers hope for patients who are resistant to chemotherapy and those with absolute contraindications to the use of platinum-based agents [72]. Targeted therapies are also within the therapeutic range, including FDA-approved erdafitinib, which is used in the treatment of advanced bladder cancer after platinum treatment progression [60]. Intensive clinical trials also prove the effectiveness of other preparations in this indication. Targeted therapy, compared with chemotherapy, has often shown a better safety profile and fewer adverse events associated with the treatment. Hyperphosphatemia conventionally occurring during treatment can be extruded through the use of novel, more specific inhibitors that reduce the side effect profile. The most severe complication of this therapy seems to be retinopathy, which must be addressed in further clinical trials in order to determine safe doses of targeted therapy. The effectiveness of FGFR inhibitors has been proven in various neoplasms in which a mutation of a given gene has been found. However we still face limitations, such as therapy resistance [88,89,90]. In light of that, it is imperative to extend the research to tumors without mutations and to evaluate the efficacy of targeted therapy in combination with immunotherapy. The first clinical trials show only the effectiveness of interfering with the tumor by these preparations; it seems necessary to precisely determine the optimal method, dose, and duration. We believe that the currently available data serve as a rationale for a therapy composed of novel, targeted therapies in combination with currently established medication. Undoubtedly, this is an impressive step forward in understanding the mechanism of tumor growth and stopping it locally, without interfering with the overall well-being of the patient.

## Figures and Tables

**Table 1:** FGFR inhibitors in urothelial carcinoma, currently under clinical trial or marked.

Medication	Target	Population	Phase	Comedication	NCT
Erdafitinib	FGFR1–3	FGFR2/3 mutation or fusion	Marked	n.a.	NCT02365597
			Ib/II	Cetrelimab	NCT03473743
Pemigatinib	FGFR1–3	FGF or FGFR alteration platinum ineligible, FGFR3 mutation or rearrangement	II	n.a.	NCT02872714
				Pembrolizumab	NCT04003610
Rogaratinib	FGFR1–4	High FGFR1 or 3 expression cisplatin ineligible	II/III	n.a.	NCT03410693
		High FGFR1 or 3 expression	IB/II	Atezolizumab	NCT03473756
Infigratinib	FGFR1–3	Altered FGFR3	III	n.a.	NCT04197986
Futibatinib	FGFR1–4	Any solid tumor and disease progression/At least one FGF/FGFR mutation	I/II	n.a.	NCT02052778
		Advanced/metastatic cancer with FGFR2 aberration	III	Gemcitabine/Cisplatin	NCT04093362
		Advanced/metastatic urothelial carcinoma with or without FGFR mutation	II	Pembrolizumab	NCT04601857
Dovitinib	FGFR1–3	Urothelial carcinoma both with mutant FGFR3 and wild-type FGFR3	II	n.a.	NCT00790426
Vofatamab	FGFR3	Stage IV locally advanced/metastatic urothelial carcinoma, FGFR3 mutation	Ib/II	Docetaxel	NCT02401542
			II	Pembrolizumab	NCT03123055
Derazantinib	FGFR1–4	Locally advanced/metastatic solid tumor with FGFR aberration	I/II	n.a.	NCT01752920

FGF (fibroblast growth factor); FGFR (fibroblast growth factor receptor); n.a. (not applicable).

## References

[1] Sung H., Ferlay J., Siegel R.L., Laversanne M., Soerjomataram I., Jemal A., Bray F. (2021). Global Cancer Statistics 2020: GLOBOCAN Estimates of Incidence and Mortality Worldwide for 36 Cancers in 185 Countries. CA Cancer J. Clin..

[2] Wojciechowska U., Didkowska J. (2020). Nowotwory Złośliwe w Polsce w 2018. Kraj. Rejestr Nowotworów.

[3] Howlader N., Noone A.M., Krapcho M., Miller D., Brest A., Yu M., Ruhl J., Tatalovich Z., Mariotto A., Lewis D.R. (2021). SEER Cancer Statistics Review, 1975–2018.

[4] Saginala K., Barsouk A., Aluru J.S., Rawla P., Padala S.A., Barsouk A. (2020). Epidemiology of Bladder Cancer. Med. Sci..

[5] Siegel R.L., Miller K.D., Jemal A. (2019). Cancer statistics, 2019. CA Cancer J. Clin..

[6] Miyazaki J., Nishiyama H. (2017). Epidemiology of urothelial carcinoma. Int. J. Urol..

[7] Leone A., Diorio G., Sexton W., Schell M., Alexandrow M., Fahey J.W., Kumar N.B. (2017). Sulforaphane for the chemoprevention of bladder cancer: Molecular mechanism targeted approach. Oncotarget.

[8] Zaghloul M.S. (2012). Bladder cancer and schistosomiasis. J. Egypt. Natl. Canc. Inst..

[9] Mostafa M.H., Sheweita S.A., O’connor P.J. (1999). Relationship between Schistosomiasis and Bladder Cancer. Clin. Microbiol. Rev..

[10] Thun M., Linet M.S., Cerhan J.R., Haiman C.A., Schottenfeld D. (2017). Cancer Epidemiology and Prevention.

[11] Cumberbatch M.G., Rota M., Catto J.W.F., La Vecchia C. (2016). The Role of Tobacco Smoke in Bladder and Kidney Carcinogenesis: A Comparison of Exposures and Meta-analysis of Incidence and Mortality Risks. Eur. Urol..

[12] Letašiová S., Medveďová A., Šovčíková A., Dušinská M., Volkovová K., Mosoiu C., Bartonová A. (2012). Bladder cancer, a review of the environmental risk factors. Environ. Health.

[13] Hinotsu S., Akaza H., Miki T., Fujimoto H., Shinohara N., Kikuchi E., Mizutani Y., Koga H., Okajima E., Okuyama A. (2009). Bladder cancer develops 6 years earlier in current smokers: Analysis of bladder cancer registry data collected by the cancer registration committee of the Japanese Urological Association. Int. J. Urol..

[14] Dinney C.P.N., McConkey D.J., Millikan R.E., Wu X., Bar-Eli M., Adam L., Kamat A.M., Siefker-Radtke A.O., Tuziak T., Sabichi A.L. (2004). Focus on bladder cancer. Cancer Cell.

[15] Jabłonowski Z. (2013). Rak pęcherza moczowego—Epidemiologia, diagnostyka i leczenie w XXI wieku. Folia Med. Lodz..

[16] Patel V.G., Oh W.K., Galsky M.D. (2020). Treatment of muscle-invasive and advanced bladder cancer in 2020. CA Cancer J. Clin..

[17] Soloway M.S., Ikard M., Ford K. (1981). Cis-diamminedichloroplatinum (II) in locally advanced and metastatic urothelial cancer. Cancer.

[18] Scher H.I., Yagoda A., Herr H.W., Sternberg C.N., Bosl G., Morse M.J., Sogani P.C., Watson R.C., Dershaw D.D., Reuter V. (1988). Neoadjuvant M-Vac (Methotrexate, Vinblastine, Doxorubicin and Cisplatin) Effect on the Primary Bladder Lesion. J. Urol..

[19] Facchini G., Cavaliere C., Romis L., Mordente S. (2020). Advanced/metastatic bladder cancer: Current status and future directions. Eur. Rev. Med. Pharmacol. Sci..

[20] Powles T., Park S.H., Voog E., Caserta C., Valderrama B.P., Gurney H., Kalofonos H., Radulović S., Demey W., Ullén A. (2020). Avelumab Maintenance Therapy for Advanced or Metastatic Urothelial Carcinoma. N. Engl. J. Med..

[21] Witjes J.A., Bruins H.M., Cathomas R., Compérat E.M., Cowan N.C., Gakis G., Hernández V., Linares Espinós E., Lorch A., Neuzillet Y. (2021). European Association of Urology Guidelines on Muscle-invasive and Metastatic Bladder Cancer: Summary of the 2020 Guidelines. Eur. Urol..

[22] Rhea L.P., Mendez-Marti S., Kim D., Aragon-Ching J.B. (2021). Role of immunotherapy in bladder cancer. Cancer Treat. Res. Commun..

[23] Warrick J.I., Knowles M.A., Yves A., van der Kwast T., Grignon D.J., Kristiansen G., Egevad L., Hartmann A., Cheng L. (2020). Report from the International Society of Urological Pathology (ISUP) Consultation Conference on Molecular Pathology of Urogenital Cancers. II. Molecular Pathology of Bladder Cancer. Am. J. Surg. Pathol..

[24] The Cancer Genome Atlas Reaserch Network (2014). Comprehensive molecular characterization of urothelial bladder carcinoma. Nature.

[25] Turner N., Grose R. (2010). Fibroblast growth factor signalling: From development to cancer. Nat. Rev. Cancer.

[26] Gui Y., Guo G., Huang Y., Hu X., Tang A., Gao S., Wu R., Chen C., Li X., Zhou L. (2011). Frequent mutations of chromatin remodeling genes in transitional cell carcinoma of the bladder. Nat. Genet..

[27] Satyal U., Sikder R.K., Mcconkey D., Plimack E.R., Abbosh P.H. (2019). Clinical implications of molecular subtyping in bladder cancer. Curr. Opin. Urol..

[28] Robertson A.G., Kim J., Al-Ahmadie H., Bellmunt J., Guo G., Cherniack A.D., Hinoue T., Laird P.W., Hoadley K.A., Akbani R. (2017). Comprehensive Molecular Characterization of Muscle-Invasive Bladder Cancer. Cell.

[29] Thisse B., Thisse C. (2005). Functions and regulations of fibroblast growth factor signaling during embryonic development. Dev. Biol..

[30] Ornitz D.M., Legeai-Mallet L. (2017). Achondroplasia: Development, pathogenesis, and therapy. Dev. Dyn..

[31] Beenken A., Mohammadi M. (2009). The FGF family: Biology, pathophysiology and therapy. Nat. Rev. Drug Discov..

[32] Kalinina J., Dutta K., Ilghari D., Beenken A., Goetz R., Eliseenkova A.V., Cowburn D., Mohammadi M. (2012). The alternatively spliced acid box region plays a key role in FGF receptor autoinhibition. Structure.

[33] Jin C., Wang F., Wu X., Yu C., Luo Y., McKeehan W.L. (2004). Directionally specific paracrine communication mediated by epithelial FGF9 to stromal FGFR3 in two-compartment premalignant prostate tumors. Cancer Res..

[34] Sleeman M., Fraser J., Mcdonald M., Yuan S., White D., Grandison P., Kumble K., Watson J.D., Murison J.G. (2001). Identification of a new fibroblast growth factor receptor, FGFR5. Gene.

[35] Trueb B. (2011). Biology of FGFRL1, the fifth fibroblast growth factor receptor. Cell. Mol. Life Sci..

[36] Goetz R., Mohammadi M. (2013). Exploring mechanisms of FGF signalling through the lens of structural biology. Nat. Rev. Mol. Cell Biol..

[37] Itoh N., Ornitz D.M. (2004). Evolution of the Fgf and Fgfr gene families. Trends Genet..

[38] Tomlinson D.C., Hurst C.D., Knowles M.A. (2007). Knockdown by shRNA identifies S249C mutant FGFR3 as a potential therapeutic target in bladder cancer. Oncogene.

[39] Teven C.M., Farina E.M., Rivas J., Reid R.R. (2014). Fibroblast growth factor (FGF) signaling in development and skeletal diseases. Genes Dis..

[40] Ornitz D.M., Itoh N. (2015). The fibroblast growth factor signaling pathway. Wiley Interdiscip. Rev. Dev. Biol..

[41] Hanahan D., Weinberg R.A. (2011). Hallmarks of cancer: The next generation. Cell.

[42] Matsumoto H., Shiraishi K., Azuma H., Inoue K., Uemura H., Eto M., Ohyama C., Ogawa O., Kikuchi E., Kitamura H. (2020). Clinical Practice Guidelines for Bladder Cancer 2019 update by the Japanese Urological Association: Summary of the revision. Int. J. Urol..

[43] El Rassy E., Assi T., Bakouny Z., Pavlidis N., Kattan J. (2019). Beyond first-line systemic treatment for metastatic urothelial carcinoma of the bladder. Clin. Transl. Oncol..

[44] Eswarakumar V.P., Lax I., Schlessinger J. (2005). Cellular signaling by fibroblast growth factor receptors. Cytokine Growth Factor Rev..

[45] Goebell P.J., Knowles M.A. (2010). Bladder cancer or bladder cancers? Genetically distinct malignant conditions of the urothelium. Urol. Oncol. Semin. Orig. Investig..

[46] Williams S.V., Hurst C.D., Knowles M.A. (2013). Oncogenic FGFR3 gene fusions in bladder cancer. Hum. Mol. Genet..

[47] Stein J.P., Lieskovsky G., Cote R., Groshen S., Feng A.-C., Boyd S., Skinner E., Bochner B., Thangathurai D., Mikhail M. (2001). Radical Cystectomy in the Treatment of Invasive Bladder Cancer: Long-Term Results in 1,054 Patients. J. Clin. Oncol..

[48] Hernández S., López-Knowles E., Lloreta J., Kogevinas M., Amorós A., Tardón A., Carrato A., Serra C., Malats N., Real F.X. (2006). Prospective Study of FGFR3 Mutations as a Prognostic Factor in Nonmuscle Invasive Urothelial Bladder Carcinomas. J. Clin. Oncol..

[49] Karoui M., Hofmann-Radvanyi H., Zimmermann U., Couvelard A., Degott C., Faridoni-Laurens L., Ahomadegbe J.-C., Gazzeri S., Brambilla E., Clerici T. (2001). No evidence of somatic FGFR3 mutation in various types of carcinoma. Oncogene.

[50] Bakkar A.A., Wallerand H., Radvanyi F., Lahaye J.-B., Pissard S., Lecerf L., Kouyoumdjian J.C., Abbou C.C., Pairon J.-C., Jaurand M.-C. (2003). FGFR3 and TP53 gene mutations define two distinct pathways in urothelial cell carcinoma of the bladder. Cancer Res..

[51] Tomlinson D.C., Baldo O., Hamden P., Knowles M.A. (2007). FGFR3 protein expression and its relationship to mutation status and prognostic variables in bladder cancer. J. Pathol..

[52] Neuzillet Y., van Rhijn B.W.G., Prigoda N.L., Bapat B., Liu L., Bostrom P.J., Fleshner N.E., Gallie B.L., Zlotta A.R., Jewett M.A.S. (2014). FGFR3 mutations, but not FGFR3 expression and FGFR3 copy-number variations, are associated with favourable non-muscle invasive bladder cancer. Virchows Arch..

[53] Pouessel D., Neuzillet Y., Mertens L.S., van der Heijden M.S., de Jong J., Sanders J., Peters D., Leroy K., Manceau A., Maille P. (2016). Tumor heterogeneity of fibroblast growth factor receptor 3 (FGFR3) mutations in invasive bladder cancer: Implications for perioperative anti-FGFR3 treatment. Ann. Oncol..

[54] Guancial E.A., Werner L., Bellmunt J., Bamias A., Choueiri T.K., Ross R., Schutz F.A., Park R.S., O’Brien R.J., Hirsch M.S. (2014). FGFR3 expression in primary and metastatic urothelial carcinoma of the bladder. Cancer Med..

[55] Foth M., Ismail N.F.B., Kung J.S.C., Tomlinson D., Knowles M.A., Eriksson P., Sjödahl G., Salmond J.M., Sansom O.J., Iwata T. (2018). FGFR3 mutation increases bladder tumourigenesis by suppressing acute inflammation. J. Pathol..

[56] Sweis R.F., Spranger S., Bao R., Paner G.P., Stadler W.M., Steinberg G., Gajewski T.F. (2016). Molecular Drivers of the Non–T-cell-Inflamed Tumor Microenvironment in Urothelial Bladder Cancer. Cancer Immunol. Res..

[57] Zhao J., Tan W., Zhang L., Liu J., Shangguan M., Chen J., Zhao B., Peng Y., Cui M., Zhao S. (2021). FGFR3 phosphorylates EGFR to promote cisplatin-resistance in ovarian cancer. Biochem. Pharmacol..

[58] Necchi A., Raggi D., Giannatempo P., Marandino L., Farè E., Gallina A., Colecchia M., Lucianò R., Salonia A., Gandaglia G. (2021). Can Patients with Muscle-invasive Bladder Cancer and Fibroblast Growth Factor Receptor-3 Alterations Still Be Considered for Neoadjuvant Pembrolizumab? A Comprehensive Assessment from the Updated Results of the PURE-01 Study. Eur. Urol. Oncol..

[59] Loriot Y., Necchi A., Park S.H., Garcia-Donas J., Huddart R., Burgess E., Fleming M., Rezazadeh A., Mellado B., Varlamov S. (2019). Erdafitinib in Locally Advanced or Metastatic Urothelial Carcinoma. N. Engl. J. Med..

[60] Perera T.P.S., Jovcheva E., Mevellec L., Vialard J., De Lange D., Verhulst T., Paulussen C., Van De Ven K., King P., Freyne E. (2017). Discovery and Pharmacological Characterization of JNJ-42756493 (Erdafitinib), a Functionally Selective Small-Molecule FGFR Family Inhibitor. Mol. Cancer Ther..

[61] Mahipal A., Tella S.H., Kommalapati A., Yu J., Kim R. (2020). Prevention and treatment of FGFR inhibitor-associated toxicities. Crit. Rev. Oncol. Hematol..

[62] Loriot Y., Necchi A., Park S.H., Huddart R.A., Burgess E., Zhong B., Santiago-Walker A., Little S., Roccia T., De Porre P. (2018). Erdafitinib compared with vinflunine or docetaxel or pembrolizumab in patients (pts) with metastatic or surgically unresectable (M/UR) urothelial carcinoma (UC) and selected fgfr gene alterations (FGFRalt): The phase III THOR study. Ann. Oncol..

[63] Necchi A., Pouessel D., Leibowitz-Amit R., Flechon A., Gupta S., Barthelemy P., Maio M., Zhu X., Asatiani E., Serbest G. (2018). Interim results of fight-201, a phase II, open-label, multicenter study of INCB054828 in patients (pts) with metastatic or surgically unresectable urothelial carcinoma (UC) harboring fibroblast growth factor (FGF)/FGF receptor (FGFR) genetic alterations. Ann. Oncol..

[64] Galsky M.D., Powles T., Dreicer R., Kitamura H., Asatiani E., Howe J., Zhen H., Oliveira N., Necchi A. (2020). FIGHT-205: Phase II study of first-line pemigatinib (PEMI) plus pembrolizumab (PEMBRO) versus PEMI alone versus standard of care (SOC) for cisplatin (CIS)—Ineligible urothelial carcinoma (UC) with FGFR3 mutation or rearrangement. J. Clin. Oncol..

[65] Grünewald S., Politz O., Bender S., Héroult M., Lustig K., Thuss U., Kneip C., Kopitz C., Zopf D., Collin M. (2019). Rogaratinib: A potent and selective pan-FGFR inhibitor with broad antitumor activity in FGFR-overexpressing preclinical cancer models. Int. J. Cancer.

[66] Quinn D.I., Petrylak D.P., Bellmunt J., Necchi A., Gurney H., Lee J.-L., Van Der Heijden M.S., Rosenbaum E., Penel N., Pang S.-T. (2020). FORT-1: Phase II/III study of rogaratinib versus chemotherapy (CT) in patients (pts) with locally advanced or metastatic urothelial carcinoma (UC) selected based on FGFR1/3 mRNA expression. J. Clin. Oncol..

[67] Rosenberg J.E., Gajate P., Morales-Barrera R., Lee J.-L., Necchi A., Penel N., Zagonel V., Sierecki M.R., Piciu A.-M., Ellinghaus P. (2020). Safety and preliminary efficacy of rogaratinib in combination with atezolizumab in a phase Ib/II study (FORT-2) of first-line treatment in cisplatin-ineligible patients (pts) with locally advanced or metastatic urothelial cancer (UC) and FGFR mRNA overexp. J. Clin. Oncol..

[68] Daneshmand S., Grivas P., Sridhar S.S., Gupta S., Bellmunt J., Sonpavde G., Fleming M.T., Lerner S.P., Loriot Y., Wang H. (2020). PROOF 302: A randomized, double-blind, placebo-controlled, phase III trial of infigratinib as adjuvant therapy in patients with invasive urothelial carcinoma harboring susceptible FGFR3 alterations. J. Clin. Oncol..

[69] Bahleda R., Meric-Bernstam F., Goyal L., Tran B., He Y., Yamamiya I., Benhadji K.A., Matos I., Arkenau H.T. (2020). Phase I, first-in-human study of futibatinib, a highly selective, irreversible FGFR1–4 inhibitor in patients with advanced solid tumors. Ann. Oncol..

[70] Goyal L., Meric-Bernstam F., Hollebecque A., Valle J.W., Morizane C., Karasic T.B., Abrams T.A., Furuse J., He Y., Soni N. (2020). FOENIX-CCA2: A phase II, open-label, multicenter study of futibatinib in patients (pts) with intrahepatic cholangiocarcinoma (iCCA) harboring FGFR2 gene fusions or other rearrangements. J. Clin. Oncol..

[71] Milowsky M.I., Dittrich C., Durán I., Jagdev S., Millard F.E., Sweeney C.J., Bajorin D., Cerbone L., Quinn D.I., Stadler W.M. (2014). Phase 2 trial of dovitinib in patients with progressive FGFR3-mutated or FGFR3 wild-type advanced urothelial carcinoma. Eur. J. Cancer.

[72] Mendiratta P., Grivas P. (2018). Emerging biomarkers and targeted therapies in urothelial carcinoma. Ann. Transl. Med..

[73] Casadei C., Dizman N., Schepisi G., Cursano M.C., Basso U., Santini D., Pal S.K., De Giorgi U. (2019). Targeted therapies for advanced bladder cancer: New strategies with FGFR inhibitors. Ther. Adv. Med. Oncol..

[74] Bellmunt J., Picus J., Kohli M., Arriaga Y.E., Milowsky M.I., Currie G., Abella S., Pal S.K. (2018). FIERCE-21: Phase 1b/2 study of docetaxel + b-701, a selective inhibitor of FGFR3, in relapsed or refractory (R/R) metastatic urothelial carcinoma (mUCC). J. Clin. Oncol..

[75] Necchi A., Castellano D.E., Mellado B., Pang S., Urun Y., Park S.H., Vaishampayan U.N., Currie G., Abella-Dominicis E., Pal S.K. (2019). Fierce-21: Phase II study of vofatmab (B-701), a selective inhibitor of FGFR3, as salvage therapy in metastatic urothelial carcinoma (mUC). J. Clin. Oncol..

[76] Siefker-Radtke A.O., Currie G., Abella E., Vaena D.A., Rezazadeh Kalebasty A., Curigliano G., Tupikowski K., Andric Z.G., Lugowska I., Kelly W.K. (2019). FIERCE-22: Clinical activity of vofatamab (V) a FGFR3 selective inhibitor in combination with pembrolizumab (P) in WT metastatic urothelial carcinoma, preliminary analysis. J. Clin. Oncol..

[77] Li J., Alyamani M., Zhang A., Chang K.-H., Berk M., Li Z., Zhu Z., Petro M., Magi-Galluzzi C., Taplin M.-E. (2017). Aberrant corticosteroid metabolism in tumor cells enables GR takeover in enzalutamide resistant prostate cancer. eLife.

[78] Kobayashi S., Boggon T.J., Dayaram T., Jänne P.A., Kocher O., Meyerson M., Johnson B.E., Eck M.J., Tenen D.G., Halmos B. (2005). EGFR Mutation and Resistance of Non–Small-Cell Lung Cancer to Gefitinib. N. Engl. J. Med..

[79] Yun C.-H., Mengwasser K.E., Toms A.V., Woo M.S., Greulich H., Wong K.-K., Meyerson M., Eck M.J. (2008). The T790M mutation in EGFR kinase causes drug resistance by increasing the affinity for ATP. Proc. Natl. Acad. Sci. USA.

[80] Turke A.B., Zejnullahu K., Wu Y.-L., Song Y., Dias-Santagata D., Lifshits E., Toschi L., Rogers A., Mok T., Sequist L. (2010). Preexistence and Clonal Selection of MET Amplification in EGFR Mutant NSCLC. Cancer Cell.

[81] Chen Z., Li Y., Tan B., Zhao Q., Fan L., Li F., Zhao X. (2020). Progress and current status of molecule-targeted therapy and drug resistance in gastric cancer. Drugs Today.

[82] Cowell J.K., Qin H., Hu T., Wu Q., Bhole A., Ren M. (2017). Mutation in the FGFR1 tyrosine kinase domain or inactivation of PTEN is associated with acquired resistance to FGFR inhibitors in FGFR1-driven leukemia/lymphomas. Int. J. Cancer.

[83] Chell V., Balmanno K., Little A.S., Wilson M., Andrews S., Blockley L., Hampson M., Gavine P.R., Cook S.J. (2013). Tumour cell responses to new fibroblast growth factor receptor tyrosine kinase inhibitors and identification of a gatekeeper mutation in FGFR3 as a mechanism of acquired resistance. Oncogene.

[84] Chatterjee N., Bivona T.G. (2019). Polytherapy and Targeted Cancer Drug Resistance. Trends Cancer.

[85] Wang L., Šuštić T., Leite de Oliveira R., Lieftink C., Halonen P., van de Ven M., Beijersbergen R.L., van den Heuvel M.M., Bernards R., van der Heijden M.S. (2017). A Functional Genetic Screen Identifies the Phosphoinositide 3-kinase Pathway as a Determinant of Resistance to Fibroblast Growth Factor Receptor Inhibitors in FGFR Mutant Urothelial Cell Carcinoma. Eur. Urol..

[86] Malchers F., Ercanoglu M., Schütte D., Castiglione R., Tischler V., Michels S., Dahmen I., Brägelmann J., Menon R., Heuckmann J.M. (2017). Mechanisms of Primary Drug Resistance in FGFR1 -Amplified Lung Cancer. Clin. Cancer Res..

[87] Shan L., Liu W., Zhan Y. (2019). Sulfated polysaccharide of Sepiella maindroni ink targets Akt and overcomes resistance to the FGFR inhibitor AZD4547 in bladder cancer. Aging.

[88] Jiang K., Tang X., Guo J., He R., Chan S., Song X., Tu Z., Wang Y., Ren X., Ding K. (2021). GZD824 overcomes FGFR1-V561F/M mutant resistance in vitro and in vivo. Cancer Med..

[89] Babina I.S., Turner N.C. (2017). Advances and challenges in targeting FGFR signalling in cancer. Nat. Rev. Cancer.

[90] Yue S., Li Y., Chen X., Wang J., Li M., Chen Y., Wu D. (2021). FGFR-TKI resistance in cancer: Current status and perspectives. J. Hematol. Oncol..

